# Supplementary material for: Triplet superconductivity in coupled odd-gon rings
Source: Sci Rep. 2019 Feb 25;9:2691. doi: 10.1038/s41598-019-39130-4 (PMC6389994; doi:10.1038/s41598-019-39130-4)
Supplement: Supplementary file 1 — Supplemental Information for [file 41598_2019_39130_MOESM1_ESM.pdf]

Supplemental Information for Triplet superconductivity in coupled odd-gon rings

Sahinur Reja[1] and Satoshi Nishimoto[2,3]

[1] Department of Physics, Indiana University, Bloomington, Indiana 47405, USA
[2] Department of Physics, TU Dresden, 01069 Dresden, Germany
[3] Institute for Theoretical Solid State Physics, IFW Dresden, 01069 Dresden, Germany

I. Transfer integrals for $A_2Cr_3As_3$ (A=K, Rb, and Cs)

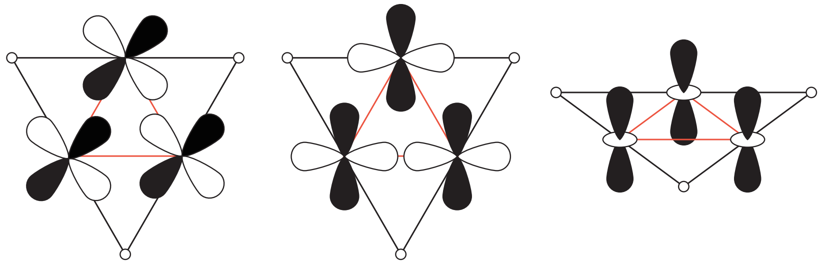

FIG. S1. Schematic pictures of chromium $3d$ orbitals dominating the conductivity in $A_2Cr_3As_3$.

In the main text, a model for $A_2Cr_3As_3$ was constructed by taking a Cr site as a Hubbard site. Therefore, we here estimate the transfer integrals between the Cr sites within the Slater-Koster (SK) parameterization [1]. As shown in Fig. S1, the intra-triangle (ab plane) conductivity is mostly generated from the direct hybridizations between Cu d_{xy} orbitals:

$$E_{xy,xy} = 3l^2m^2V_{dd\sigma} + (l^2 + m^2 - 4l^2m^2)V_{dd\pi} + (n^2 + l^2m^2)V_{dd\delta}, \tag{S1}$$

and between Cu $d_{x^2-y^2}$ orbitals:

$$E_{x^2-y^2,x^2-y^2} = \frac{3}{4}(l^2 - m^2)^2V_{dd\sigma} + [l^2 + m^2 - (l^2 - m^2)^2]V_{dd\pi}, \tag{S2}$$

where $V_{dd\sigma}$, $V_{dd\pi}$, and $V_{dd\delta}$ are the bond integrals for σ, π, and δ bonds, respectively. The interatomic vector is expressed as $\vec{r}_{i,j} = (r_x, r_y, r_z) = d(l, m, n)$, where d is the distance between the atoms and l, m, and n are the direction cosines to the neighboring atom. By the Muffin-Tin Orbital theory and pseudopotential theory, the bond integral is obtained as

$$V_{ddn} = \eta_{ddn}\frac{\hbar r_d^3}{md^5}, \tag{S3}$$

where r_d is a characteristic length of transition metal; it is 0.90Åfor Cr, and $\eta_{dd\sigma} = -\frac{45}{\pi}$, $\eta_{dd\pi} = \frac{30}{\pi}$, and $\eta_{dd\delta} = -\frac{15}{2\pi}$ [2]. Using the crystal structure determined by the X-ray diffraction [3], we obtain

$$E_{xy,xy} + E_{x^2-y^2,x^2-y^2} = -1.194\frac{\hbar r_d^3}{md^5} \tag{S4}$$

in electron notation, namely, the intra-triangle transfer integral in hole notation is $1.194\frac{\hbar r_d^3}{md^5} > 0$. This corresponds to t_1 in the main text. Whereas, the transfer integrals along the c-axis, t_2, is estimated by the hybridization between Cr $d_{3z^2-r^2}$ orbitals:

$$E_{3z^2-r^2,3z^2-r^2} = [n^2 - \frac{1}{2}(l^2 + m^2)]^2V_{dd\sigma} + 3n^2(l^2 + m^2)V_{dd\pi} + \frac{3}{4}(l^2 + m^2)^2V_{dd\delta} \tag{S5}$$

This value may be a few times larger than t_1. So we present the phase diagram for $t_2 = 0$ to $2t_1$ in the main text.

II. Stabilization of ferromagnetism on the on-site Coulomb repulsion

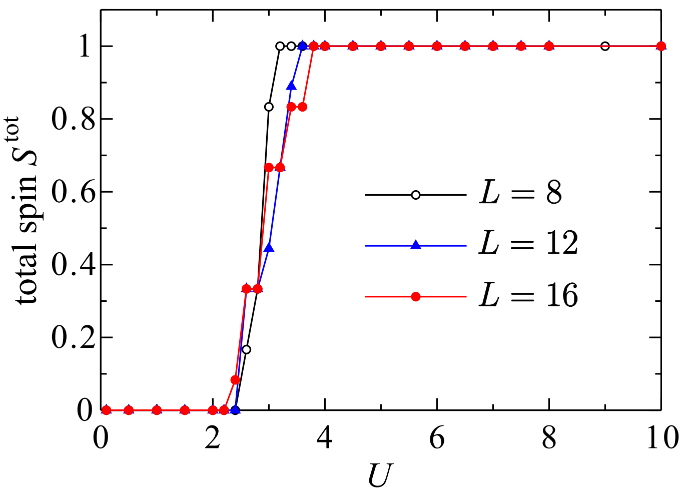

FIG. S2. Renormalized total spin as a function of the on-site Coulomb interaction for the twisted Hubbard model at $t_2 = 0.05$ and $n = 1/2$ for different clusters $L \times 3$.

To illustrate the effect of on-site Coulomb interaction U on the global ferromagnetic ordering in the twisted Hubbard model, we show the total spin as a function of U at $t_2 = 0.05$ and $n = 1/2$ in Fig. S2. We can see that the fully polarized state is stabilized for larger U, where the ferromagnetic interaction on each odd-gon, namely, J_{eff} in our effective model, is stronger. The ferromagnetic interaction is estimated from the energy difference between the spin-triplet ground state and first spin-singlet excited state of the isolated triangle including two fermions. This interaction saturates quickly with increasing U, as seen in Fig. 1 (c) of the main text, and the global ferromagnetic ordering can be stabilized even by relatively small U.

III. Coulomb repulsion in the effective model

In the main text we introduced an effective model to describe the ferromagnetism and spin-triplet superconductivity of the twisted triangular Hubbard tube. The Coulomb repulsion works when the effective site including two orbitals is occupied by three fermions. The Coulomb repulsion is roughly estimated from the single-particle gap of the isolated odd-gon, namely, $U_{\text{eff}} = (E_3 - E_2) - (E_2 - E_1)$ where E_N is the ground-state energy of the odd-gon Hubbard ring with N fermions. For example, in the case of $l_o = 3$, $E_1 = -t$, $E_2 = -2t$, and E_3 can be obtained by solving an equation $E_3^3 - 2UE_3^2 + (U^2 - 9t^2)E_3 + 6Ut^2 = 0$ where U and t are the on-site Coulomb interaction and hopping integral in the original triangle.

IV. Conductive networks of $(\text{TMTSF})_2\text{X}$, Sr_2RuO_4 and $\text{Na}_{0.35}\text{CoO}_2{\cdot}1.3\text{H}_2\text{O}$

(TMTSF)$_2$X

The crystal structure of the Bechgaard salts $(\text{TMTSF})_2\text{X}$ consists of well-separated sheets containing one-dimensional TMTSF stacks along the a-axis. The sheets are in the ab-plane and the transfer integrals along the b-axis are about 10 - 20% of those along the a-axis [4]. The unique structure of the transfer integrals can be regarded as an anisotropic triangular lattice. There are three electrons in the two highest-occupied molecular orbitals of a dimerized molecules, e.g., $(\text{TMTSF})_2$, and the system is at $\frac{3}{4}$-filling in terms of electrons, which corresponds to $\frac{1}{4}$-filling in terms of holes [5].

Sr_2RuO_4

The ruthenate Sr_2RuO_4 is a tetragonal, layered perovskite system of stacking RuO_2-planes. Like Cu in the high-T_c superconductors, Ru atoms form a square lattice. If a single-band description of RuO_2-plane for so-called γ band could be adequate, the system is described as a 2D Hubbard model with next-nearest-neighbor hopping. The next-nearest-neighbor transfer integral has been estimated to be $0.3 - 0.4$ in units of the nearest-neighbor transfer integral [6, 7], so that Ru indeed forms a triangular network. Based on the quantum oscillation measurement, the γ Fermi surface sheet is a large electron-like cylinder with the electron filling $n \sim 2/3$.

$Na_{0.35}CoO_2{\cdot}1.3H_2O$

In the cobalt oxide $Na_{0.35}CoO_2{\cdot}1.3H_2O$, the conductive *ab* planes consist of edge-sharing CoO_6 octahedra and each plane is strongly separated by Na^+ ions and H_2O molecules along *c* axis. The Co ions form a triangular lattice, and the system may be regarded as a two-dimensional triangular lattice doped with 35% electrons.

V. 2D lattice as a coupled odd-gons

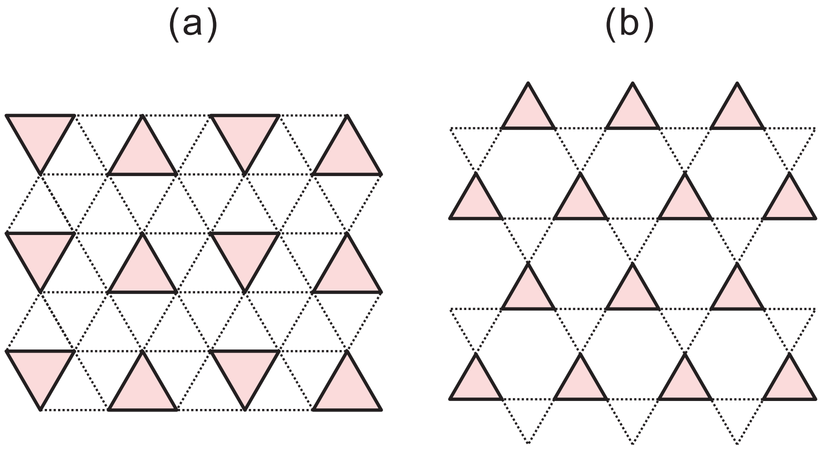

FIG. S3. (a) Triangular and (b) kagome lattices as coupled triangles. Bold and dotted lines denote intra- and inter-triangle couplings, respectively.

Many lattices can be described as coupled odd-dons. As examples, triangular and kagome lattices are illustrated in Fig. S3(a) and (b), respectively. Note that the geometry of inter-triangle couplings in not unique in the triangular lattice. The other examples of coupled odd-gons are the shastry-Sutherland, pyrochlore lattices, and fullerenes, etc. As stated in the main text, a macroscopic ferromagnetism is expected to occur in the weak inter-odd-gon-coupling limit. It should be studied in future whether a spin-triplet superconductivity appears when the ferromagnetism is melted by increasing inter-odd-gon coupling.

VI. Finite-size scaling analysis of the bonding energy

The binding energy of two fermions is defined as $\Delta_{\rm B} = \lim_{L\to\infty} \Delta_{\rm B}(L)$ with $\Delta_{\rm B}(L) = -[E_0(N_\uparrow \pm 1, N_\downarrow \pm 1; L) + E_0(N_\uparrow, N_\downarrow; L) - 2E_0(N_\uparrow \pm 1, N_\downarrow; L)]$, where $E_0(N_\uparrow, N_\downarrow; L)$ is the ground-state energy of the system with length L containing $N_\uparrow$ spin-up and $N_\downarrow$ spin-down fermions. Since this quantity has a meaning only after being extrapolated to the thermodynamic limit $L \to \infty$, the finite-size scaling analysis is necessary. In Fig. S4, some examples of the finite-size scaling analysis are shown.

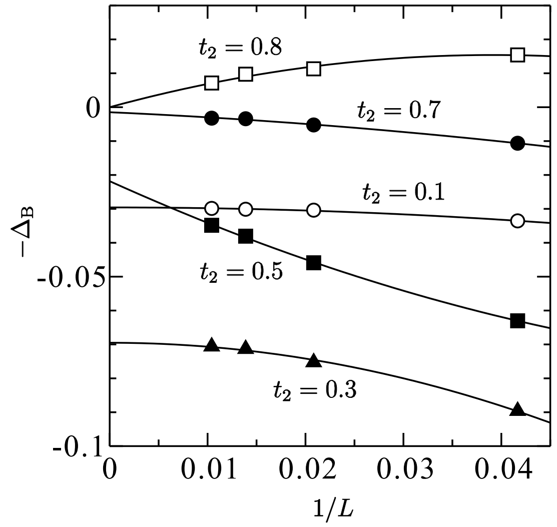

FIG. S4. Finite-size scaling analyses of the binding energy at $n = 7/12$.

VII. Superconducting pairing symmetry of $A_2Cr_3As_3$

We investigated the pairing symmetry of our triplet superconductivity in the twisted triangular Hubbard tube. Symmetry of Cooper pairs give an invaluable information of our elucidating a superconducting mechanism. So far, the possibilities of f-wave and p_z-wave pairing symmetries have been suggested for the superconductivity in $K_2Cr_3As_3$. Our DMRG calculations were performed in real space. Therefore, it is more convenient to discuss the pairing symmetry in real space. Phenomenologically, the pairing Hamiltonian for one-dimensional system is written as

$$\mathcal{H}_{\rm pair} = \frac{1}{2N} \sum_{k,k',q,\sigma,\sigma'} V_{\sigma,\sigma'}(k,k') c^\dagger_{k+q\sigma} c^\dagger_{-k\sigma'} c_{-k'\sigma'} c_{k'+q\sigma}, \tag{S6}$$

where $V_{\sigma,\sigma'}(k,k')$ is the attractive interaction. For the p-wave superconductivity, the interaction is given by

$$V_{\sigma,\sigma'}(k,k') = -V(\sqrt{2}\sin k)(\sqrt{2}\sin k'), \tag{S7}$$

where V is an averaged value of $V_{\sigma,\sigma'}(k,k')$ around the Fermi level. Following the BCS theory, we apply a mean-field approximation to Eq.(S6). It leads to

$$\mathcal{H}^{\rm MF}_{\rm pair} \simeq \frac{1}{2N} \sum_{k,k',q,\sigma,\sigma'} V_{\sigma,\sigma'}(k,k') \langle c_{-k'\sigma'} c_{k'+q\sigma}\rangle c^\dagger_{k+q\sigma} c^\dagger_{-k\sigma'} + \langle c^\dagger_{k+q\sigma} c^\dagger_{-k\sigma'}\rangle c_{-k'\sigma'} c_{k'+q\sigma} - \langle c^\dagger_{k+q\sigma} c^\dagger_{-k\sigma'}\rangle \langle c_{-k'\sigma'} c_{k'+q\sigma}\rangle. \tag{S8}$$

By usage, we define the superconducting order parameter (or gap function) as

$$\Delta_{\sigma,\sigma'}(k,q) = \frac{1}{N} \sum_{k'} V_{\sigma,\sigma'}(k,k') \langle c_{-k'\sigma'} c_{k'+q\sigma}\rangle \tag{S9}$$

Using Eq.(S9), the Hamiltonian (S8) is rewritten as

$$\mathcal{H}^{\rm MF}_{\rm pair} = \frac{1}{2} \sum_{k,q,\sigma,\sigma'} [\Delta_{\sigma,\sigma'}(q)\sin k + h.c.] + \frac{N}{4V} \sum_{q\sigma\sigma'} |\Delta_{\sigma\sigma'}(q)|^2 \tag{S10}$$

with

$$\Delta_{\sigma,\sigma'}(q) = -\frac{2V}{N} \sum_{k'} \sin k' \langle c_{-k'\sigma'} c_{k'+q\sigma}\rangle \tag{S11}$$

The Fourier transform of Eq.(S10) gives

$$\mathcal{H}^{\rm MF}_{\rm pair} = \sum_{r\sigma\sigma'} [\Delta_{\sigma\sigma'}(r) \frac{1}{2i} (c^\dagger_{r\sigma} c_{r+\hat{z}\sigma'} - c^\dagger_{r\sigma} c_{r-\hat{z}\sigma'}) + h.c.] + \frac{1}{4V} \sum_{r\sigma\sigma'} |\Delta_{\sigma\sigma'}(r)|^2 \tag{S12}$$

with the real-space order parameter

$$\Delta_{\sigma\sigma'}(r) = -iV(\langle c_{r+\hat{z}\sigma'} c_{r\sigma}\rangle - \langle c_{r-\hat{z}\sigma'} c_{r\sigma}\rangle) \tag{S13}$$

In order to determine the symmetry numerically, we calculated the pair field correlation function $D(i,j) = \langle \Delta_i \Delta_j^\dagger \rangle$ with $\Delta_i^\dagger = c_{i1\uparrow}^\dagger c_{i2\downarrow}^\dagger + c_{i1\downarrow}^\dagger c_{i2\uparrow}^\dagger$, where $c_{i\lambda\sigma}^\dagger$ creates a hole of spin σ on a site $\lambda = 1$ or 2 of the bond indexed by i. The results are shown in Fig. 5 of the main text. From Eq.(S13), $D(i,j)$ changes its sign alternately along the z-axis in the p_z pairing symmetry.

[1] J.C. Slater, G.F. Koster, Phys. Rev. **94**, 1498 (1954).
[2] W.A. Harrison: Electronic Structure and Properties of Solids (Freeman, San Francisco, 1980).
[3] J.-K. Bao, J.-Y. Liu, C.-W. Ma, Z.-H. Meng, Z.-T. Tang, Y.-L. Sun, H.-F. Zhai, H. Jiang, H. Bai, C.-M. Feng, Z.-A. Xu, and G.-H. Cao, Phys. Rev. X **5**, 011013 (2015).
[4] L. Ducasse, M. Abderrabba, J. Hoarau, M. Pesquer, B. Gallois, and J. Gaultier , J. Phys. C, **19**, 3805 (1986).
[5] D. Jérome: Organic Conductors, ed. J.P. Farges (Dekker, New York, 1994).
[6] I. Mazin and D.J. Singh, Phys. Rev. Lett. **79**, 733 (1997).
[7] A. Liebsch and A. Lichtenstein, Phys. Rev. Lett. **84**, 1591 (2000).
